# Genome-wide identification of genes with amplification and/or fusion in small cell lung cancer

**DOI:** 10.1002/gcc.22076

**Published:** 2013-05-28

**Authors:** Reika Iwakawa, Masataka Takenaka, Takashi Kohno, Yoko Shimada, Yasushi Totoki, Tatsuhiro Shibata, Koji Tsuta, Ryo Nishikawa, Masayuki Noguchi, Aiko Sato-Otsubo, Seishi Ogawa, Jun Yokota

**Affiliations:** 1 Division of Multistep Carcinogenesis, National Cancer Center Research InstituteTokyo, Japan; 2 Department of Obstetrics and Gynecology, The Jikei University School of MedicineTokyo, Japan; 3 Division of Genome Biology, National Cancer Center Research InstituteTokyo, Japan; 4 Division of Cancer Genomics, National Cancer Center Research InstituteTokyo, Japan; 5 Division of Pathology and Clinical Laboratories, National Cancer Center HospitalTokyo, Japan; 6 Department of Neuro-Oncology/Neurosurgery, International Medical Center, Saitama Medical UniversitySaitama, Japan; 7 Department of Pathology, Faculty of Medicine, University of TsukubaIbaraki, Japan; 8 Cancer Genomics Project, Tokyo UniversityTokyo, Japan

## Abstract

To obtain a landscape of gross genetic alterations in small cell lung cancer (SCLC), genome-wide copy number analysis and whole-transcriptome sequencing were performed in 58 and 42 SCLCs, respectively. Focal amplification of known oncogene loci, *MYCL1* (1p34.2), *MYCN* (2p24.3), and *MYC* (8q24.21), was frequently and mutually exclusively detected. *MYCL1* and *MYC* were co-amplified with other regions on either the same or the different chromosome in several cases. In addition, the 9p24.1 region was identified as being amplified in SCLCs without amplification of *MYC* family oncogenes. Notably, expression of the *KIAA1432* gene in this region was significantly higher in *KIAA1432* amplified cells than in non-amplified cells, and its mRNA expression showed strong correlations with the copy numbers. Thus, *KIAA1432* is a novel gene activated by amplification in SCLCs. By whole-transcriptome sequencing, a total of 60 fusion transcripts, transcribed from 95 different genes, were identified as being expressed in SCLC cells. However, no in-frame fusion transcripts were recurrently detected in ≥2 SCLCs, and genes in the amplified regions, such as *PVT1* neighboring *MYC* and *RLF* in *MYCL1* amplicons, were recurrently fused with genes in the same amplicons or with those in different amplicons on either the same or different chromosome. Thus, it was indicated that amplification and fusion of several genes on chromosomes 1 and 8 occur simultaneously but not sequentially through chromothripsis in the development of SCLC, and amplification rather than fusion of genes plays an important role in its development.

## INTRODUCTION

Lung cancer is the leading cause of cancer death worldwide, and accounts for 18% of total cancer deaths in a year (Jemal et al., 2011). In particular, most of small cell lung cancer (SCLC) cases are diagnosed after metastatic spread of the diseases, and only 5% of SCLC patients survive beyond 5 years after diagnosis (Worden and Kalemkerian, 2000; Cooper and Spiro, 2006). Therefore, for the improvement of patients’ outcome in this disease, it is necessary to identify druggable targets that are activated by genetic alterations in SCLC cells. However, since only a limited fraction of SCLC cases are treated by surgery and most of them are treated by chemotherapy and/or radiotherapy, tumor tissues are rarely available for molecular analysis. For this reason, only a few activating genetic alterations have been identified to date in SCLC cells, including amplification of the *MYC* family oncogenes, *MYCL1* (1p34), *MYCN* (2p24), and *MYC* (8q24) (Wistuba et al., 2001). Recently, whole-genome profiling has been applied to further obtain information about copy number alterations, point mutations, and fusions in SCLCs (Kim et al., 2006; Campbell et al., 2008; Pleasance et al., 2010; Voortman et al., 2010; Dooley et al., 2011; Peifer et al., 2012; Rudin et al., 2012). The results indicated that copy number gains occur in various chromosomal regions, including the *JAK2* (9p24), *FGFR1* (8p12), *TNFRSF4* (1p36), *DAD1* (14q11), *BCL2L1* (20q11), *BCL2L2* (14q11), *FAK* (8q24), *NF1B* (9p23), and *SOX2* (3q26) genes, in SCLCs. As for the gene fusions, the *PVT1* gene that is immediately downstream of the *MYC* gene at 8q24 and the *CHD7* gene at 8q12 with copy number alterations were found to be fused in the H2171 and Lu135 cell lines (Campbell et al., 2008; Pleasance et al., 2010). However, since most genetic studies in SCLC have been done using cultured cell lines, genetic alterations accumulated in fresh SCLCs *in vivo* are still unclear.

In this study, to obtain a landscape of gross genetic alterations in both fresh tumors and cell lines, genome-wide copy number analysis was performed for 33 fresh tumors and 25 cell lines to identify genes amplified in SCLCs. In parallel, whole-transcriptome sequencing was performed for 19 fresh tumors and 23 cell lines to identify fusion genes expressed in SCLCs. By copy number analysis, a novel chromosomal region amplified in a mutually exclusive manner with *MYC* family genes was identified, and genes overexpressed accompanied by gene amplification in this region was further identified. By combining the results of copy number analysis with those of whole-transcriptome sequencing, it was further revealed that fusion transcripts were often expressed from genes in several amplified regions, suggesting that amplification and fusion of genes occur simultaneously but not independently by chromothripsis in the development of SCLC.

## MATERIALS AND METHODS

### Patients and Tissues

Sixty-two tumors and corresponding non-cancerous tissues were obtained at surgery or autopsy from 1985 to 2010 at the National Cancer Center Hospital, Tokyo, Saitama Medical University, Saitama, and University of Tsukuba, Ibaraki, Japan (Supporting Information Table S1A). Genomic DNA was extracted with a QIAamp DNA mini kit (Qiagen, Hilden, Germany). Total RNA was extracted using TRIzol reagent (Invitrogen, Carlsbad, CA), purified by an RNeasy kit (Qiagen), and reverse-transcribed to cDNA by using the SuperScript III First-Strand Synthesis System (Invitrogen) with random hexamers according to the manufacturer’s instructions. This study was performed under the approval of the Institutional Review Board of the National Cancer Center.

### Cell Lines

Twenty-five cell lines were used in this study (Supporting Information Table S1B). HCC33, N417, H69, H82, H1607, H1963, H2107, H2141, and H2171 were obtained from Dr. J. D. Minna (University of Texas Southwestern, Dallas), H526 and H841 from Dr. C. C. Harris (NCI, Bethesda), Ms18 from Dr. E. Shimizu (Tottori University, Tottori, Japan), and Lu-series from Dr. T. Terasaki (National Cancer Center, Tokyo, Japan).  Other cell lines were obtained from the American Type Culture Collection or the Japanese Collection of Research Bioresources. Genomic DNA was extracted as described previously (Iwakawa et al., 2011). Poly-A(+) RNA was extracted with a Fast Track mRNA isolation kit (Invitrogen) and reverse-transcribed to cDNA as described above.

### Genome-wide Copy Number Analysis

Copy number analysis was performed using SNP-Chips for human 250K Nsp SNP arrays (Affymetrix, Inc., Santa Clara, CA). Methods used for the analysis were previously described (Nakanishi et al., 2009; Iwakawa et al., 2011). Copy numbers were determined using the Copy Number Analyzer for Affymetrix GeneChip Mapping Array (CNAG) software (Nannya et al., 2005; Yamamoto et al., 2007).

### Whole-transcriptome Sequencing

cDNA libraries for RNA sequencing were prepared using the mRNA-Seq Sample Prep Kit (Illumina, San Diego, CA) according to the manufacturer’s protocol. Briefly, poly-A(+) RNA purified from 4 μg of total RNA extracted from tumors or 0.1 μg of poly-A(+) RNA extracted from cell lines was fragmented in a fragmentation buffer, and used for double-stranded cDNA synthesis. After ligation of the paired-end (PE) adapter, a fraction of 300–350 bp was gel-purified and amplified with PCR. The resulting libraries were subjected to the PE sequencing of 50-bp reads on the Genome Analyzer IIx (GAIIx) (Illumina).

### Detection of Fusion Transcripts

PE reads derived from fusion transcripts were searched for as recently described (Kohno et al., 2012). Briefly, PE reads were mapped on human reference RNA sequences deposited in the RefSeq database using the BOWTIE program (version 0.12.5), and PE reads in which both reads were mapped on different RNA sequences were assembled to “clusters”. Paired-clusters consisting of ≥10 PE reads in each sample, for which PE reads did not appear in any of three non-cancerous lung tissues, were picked up. Paired-clusters mapped within a gene region or a neighboring-gene region (≥100 kb in the genome and the same strand) were removed due to the possibility of alternative splicing and read-through transcription. Junction reads encompassing the fusion boundaries were searched using the MapSplice (version 1.14.1) software with modifications. Transcripts that were supported by ≥10 PE reads and ≥10 junction reads were defined as gene fusions.

### Reverse Transcription (RT)-PCR and Sanger Sequencing

cDNA was amplified by PCR using KAPA Taq DNA Polymerase (KAPA Biosystems, Woburn, MA). PCR products were directly sequenced in both directions using the BigDye Termination kit and an ABI 3130xl DNA Sequencer (Applied Biosystems, Foster City, CA).

### Real-time RT-/Genomic-PCR

The amount of mRNA was quantified using TaqMan Gene Expression Assays (Applied Biosystems). The copy number of gene was determined by TaqMan Copy Number Assay (Applied Biosystems). Primers are listed in Supporting Information Table S1C. *HPRT1* and *RPPH1* were used as references for mRNA and copy number analyses, respectively. Real-time PCR was performed using the ABI 7900HT real-time PCR system (Applied Biosystems). Data was analyzed by ABI RQ Manager v1.2 for mRNA analysis and ABI Prism 7900HT Sequence Detection Software v2.3 for copy number analysis.

### Microarray Experiments and Data Processing

Two micrograms of total RNA were labeled using a 5X MEGAscript T7 kit (Ambion, Inc., Austin, Texas) and analyzed by U133Plus2.0 arrays (Affymetrix), and data was processed by the MAS5 algorithm as described previously (Okayama et al., 2012).

## RESULTS

### Amplified Genes Identified by Genome-wide Copy Number Analysis

A total of 58 SCLCs, consisted of 33 fresh tumors and 25 cell lines (Supporting Information Table S1A, B), were subjected to 250K SNP array analysis, and all genomic regions with ≥ 5 copies in ≥ 5 consecutive SNP loci were first picked up as the amplified regions in the SCLC genomes. However, by these criteria, whole chromosomes or whole chromosomal arms were more frequently picked up than focal chromosomal regions in various chromosomes among various tumors and cell lines. Therefore, amplified chromosomal regions defined as segments of ≥5 consecutive SNP loci with estimated copy numbers of ≥6 were next picked up from each SCLC. Ten amplified regions were identified on chromosomes 1p, 8q, 9p, 12p, and 19p in 7 of the 33 fresh tumors (Supporting Information Table S2). Sizes of amplified regions ranged from 0.05 to 3.61 Mb (mean ± SD = 1.06 ± 1.25 Mb), and 110 genes were mapped in these regions. Forty-seven amplified regions were identified on chromosomes 1p, 2p, 8q, 9p, 12p, 14q, 17q, and 20q in 13 of the 25 cell lines (Supporting Information Table S2). Sizes of amplified regions ranged from 0.08 to 4.22 Mb (mean ± SD = 0.67 ± 0.81 Mb), and 211 genes were mapped in these regions. Therefore, various chromosomal regions were identified as being focally amplified by the criteria of copy numbers ≥6, sizes of amplified regions were similar in fresh tumors and cell lines, and the several amplified regions in fresh tumors overlapped with those in cell lines (Supporting Information Tables S2). Accordingly, commonly amplified regions were determined by comparison of amplified regions among all the 58 SCLCs, including both fresh tumors and cell lines. Eight regions on chromosomes 1p, 2p, 8q, and 9p were commonly (≥2 SCLCs) amplified in these SCLCs (Table 1). Sizes of the regions ranged from 0.03 to 0.77 Mb (mean ± SD = 0.25 ± 0.23 Mb), and 34 genes were mapped in these regions. Three of the 8 regions contained *MYC* family oncogenes, *MYCL1*, *MYCN,* and *MYC*, respectively, known to be frequently amplified in SCLCs (Wistuba et al., 2001; Kim et al., 2006; Voortman et al., 2010; Larsen and Minna, 2011).

**TABLE 1:** Chromosomal Regions and Genes Commonly Amplified in Small Cell Lung Cancers

						Sample name		
No	Cytoband	Start (Mb)	End (Mb)	Size (Mb)	Gene	Fresh tumor	Cell line	No. of amplified SCLCs
1	1p34.3	37.37	38.19	0.77	*LOC728431, ZC3H12A, MEAF6, SNIP1, DNALI1, GNL2, RSPO1, C1orf109, CDCA8, EPHA10, MANEAL, YRDC, C1orf122, MTF1, INPP5B, SF3A3, FHL3, UTP11L, POU3F1*	SM09-008T	H1184	
							H510	
							3
2	1p34.2	39.96	40.05	0.09	*TRIT1, MYCL1*	SM09-012T	H1184	6
							H1963	
							H510	
							HCC33	
							H2141	
3	2p24.3	15.98	16.16	0.18	*MYCNOS, MYCN*	-	H526	2
							H69	
4	8q12.2	62.01	62.18	0.17	*LOC100130298*	-	H2171	3
							Lu135	
							N417	
5	8q12.2	62.36	62.39	0.03	*CLVS1*	-	H2171	3
							Lu135	
							N417	
6	8q24.21	128.75	128.88	0.13	*MYC, MIR1204, PVT1*		H2171	6
						SM09-011T1	H446	
						SM09-019T	H82	
							N417	
7	9p24.1	5.35	5.78	0.43	*PLGRKT, CD274, PDCD1LG2*, **KIAA1432**, *ERMP1*	R-513T	H1607	3
						SM09-010T		
8	9p23	13.27	13.45	0.18	*FLJ41200*	-	H1607	2
							H446	

Candidate target genes are described in bold.

*MYCL1* and *MYCN* were co-amplified with *TRIT1* at 1p34.2 and *MYCNOS* at 2p24.3, respectively, in 6 and 2 SCLCs. In the 8q24.21 region, *MYC*, *MIR1204,* and *PVT1* were co-amplified in 6 SCLCs. Copy number breakpoints of amplified regions at 8q24.21 were mapped in the *PVT1* gene in five of the six SCLCs (Supporting Information Tables S2; Supporting Information Fig. S1). The 1p34.3 region was co-amplified with *MYCL1* at 1p34.2, and the 8q12.2 regions were co-amplified with *MYC* at 8q24.21, respectively, in several SCLCs (Supporting Information Fig. S2). Therefore, occurrence of complicated intrachromosomal rearrangements was suggested in the process of *MYCL1* and *MYC* amplification, resulting in the co-amplification of several other genes on chromosomes 1 and 8, respectively.

**Figure 1:**
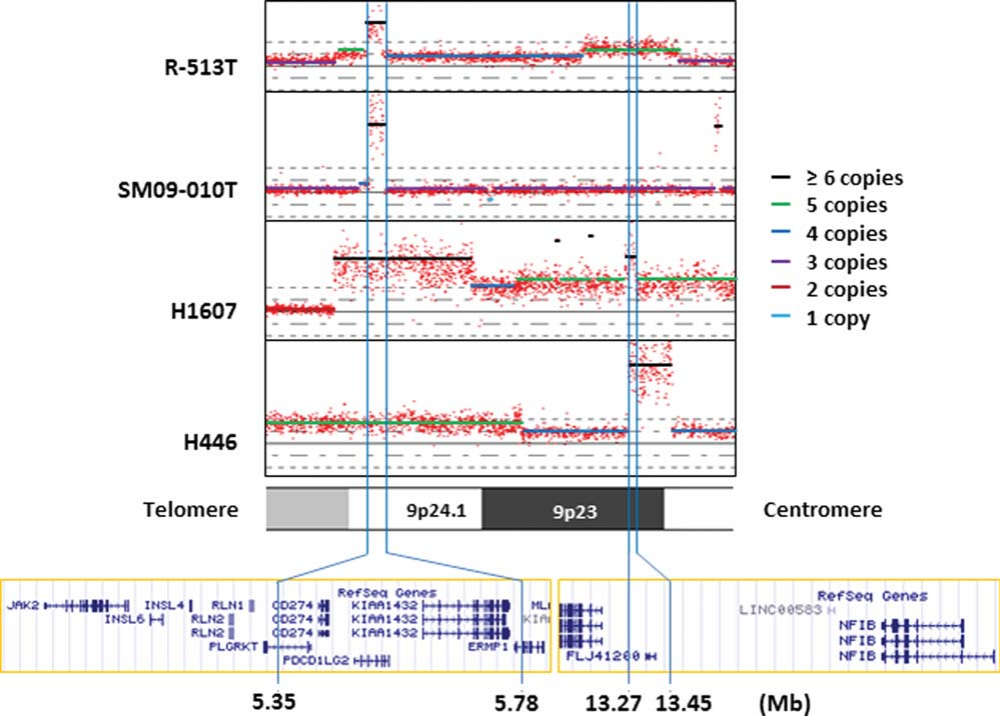
Copy number plots of commonly amplified regions on chromosome 9p in four SCLCs. Copy numbers determined by 250K SNP array analysis are indicated by bars in colors. Genes mapped in two commonly amplified regions were aligned according to the BLAST human sequences (Build 37.3) in the NCBI database (http://www.ncbi.nlm.nih.gov/).

In addition to the regions on chromosomes 1, 2, and 8, two novel commonly amplified regions were identified on chromosome 9p, 9p23, and 9p24.1 (Table 1; Fig. 1). The 9p23 region including *FLJ41200* was amplified in 2 SCLCs, whereas the 9p24.1 region, including *PLGRKT*, *CD274*, *PDCD1LG2*, *KIAA1432,* and *ERMP1*, was amplified in three SCLCs. Both regions were co-amplified in the H1607 cell line, whereas only the 9p23 region was amplified in the H446 cell line and only the 9p24.1 region was amplified in two fresh tumors (Supporting Information Fig. S2). On chromosome 9p, *NFIB* at 9p23 and *JAK2* at 9p24.1 were reported to be amplified in SCLC (Voortman et al., 2010; Dooley et al., 2011). However, *NFIB* and *JAK2* were amplified only in one SCLC, respectively. Therefore, these two genes were not mapped in the commonly amplified regions on chromosome 9p (Supporting Information Table S2; Table 1; Fig. 1).

### Expression of Amplified Genes on Chromosome 9p

Five genes, *PLGRKT*, *CD274*, *PDCD1LG2*, *KIAA1432,* and *ERMP1,* were mapped in the commonly amplified region at 9p24.1 (Table 1). To determine which genes were overexpressed by gene amplification, their mRNA expression was profiled in 19 fresh tumors (Supporting Information Fig. S3A). These five genes were amplified in one of the 19 tumors, SM09-010T. Expression of *PLGRKT*, *CD274* and *KIAA1432*, but not of *PDCD1LG2* and *ERMP1,* was distinctly high in SM09-010T (Supporting Information Fig. S3B). Therefore, *PLGRKT*, *CD274,* and *KIAA1432* could be overexpressed by gene amplification in SCLCs.

**Figure 2:**
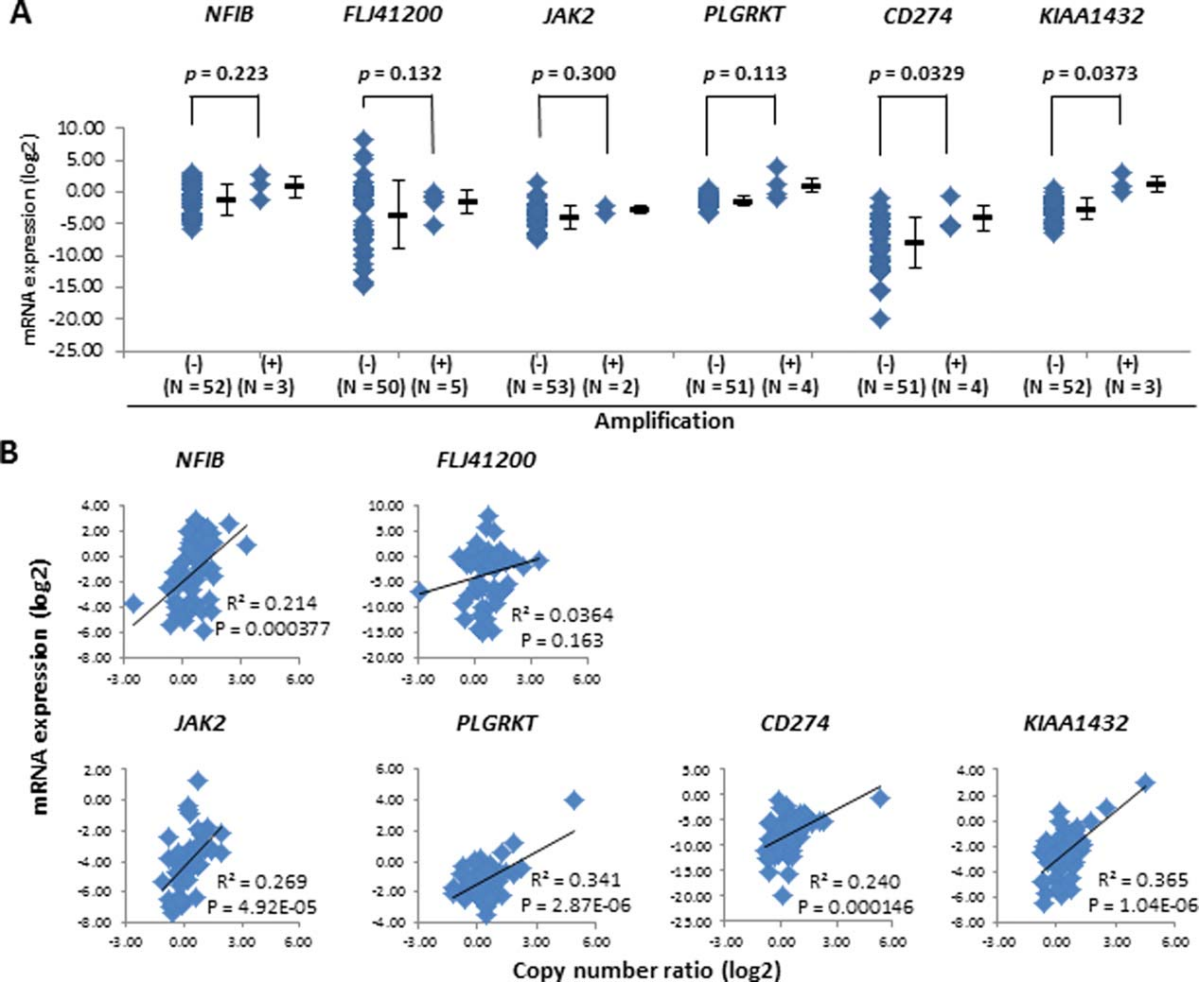
Association of copy numbers with expression levels in the 9p23-24 genes in SCLC cells. Levels of mRNA expression were quantified as ΔCt values using the *HPRT1* gene as a control. Levels relative to normal lung were calculated using Human Lung Poly-A(+) RNA (Clontech) as the calibrator. (A) Levels of mRNA expression (log2) quantified by real-time RT-PCR in amplified (+) and not amplified (–) SCLCs. *P*-values by Student’s *T*-test for differences are shown. (B) Correlation of copy number ratios by real-time genomic-PCR with mRNA expression levels by real-time RT-PCR among 55 SCLCs.

To further determine genes whose expression is associated with copy numbers on chromosome 9p, we next performed the association study of gene expression with gene copy number in 55 SCLCs, including 30 fresh tumors and 25 cell lines (Supporting Information Table S1A, B). In addition to *PLGRKT*, *CD274* and *KIAA1432* at 9p24.1 and *FLJ41200* at 9p23 as genes commonly amplified in SCLCs, *NFIB* at 9p23 and *JAK2* at 9p24.1 were also subjected to the analysis. Expression of *CD274* and *KIAA1432* in amplified cells was significantly higher than that in non-amplified cells (*P* < 0.05) (Fig. 2A), and five genes except *FLJ41200* showed significant associations between the levels of mRNA expression and copy numbers (*P* < 0.05) (Fig. 2B). Notably, *KIAA1432* showed the strongest association between them (*P* = 1.04E-06). Therefore, *KIAA1432* is the strongest target activated by gene amplification on chromosome 9p in SCLC. If there is another target in the 9p23 region, *NF1B* is more likely to be the one than *FLJ41200*.

### Copy Numbers of Amplified Genes Defined by Real-time Genomic-PCR

To further investigate the prevalence and specificity of gene amplification in the chromosome 1, 2, 8, and 9 regions in SCLCs, 87 SCLCs comprised of 62 tumors and 25 cell lines were subjected to real-time genomic-PCR analyses. Among them, 33 tumors and 25 cell lines were also subjected to 250K SNP array analyses (Supporting Information Table S1A, B). Three *MYC* family genes and six genes on chromosome 9p were analyzed, and criteria of gene amplification by real-time genomic-PCR were defined as DNA copy number ratios ≥3 that was equivalent to the copy number ≥6. Five of the 33 tumors and 12 of the 25 cell lines showed amplification of 1–5 of the 9 genes by 250K SNP array analysis (Supporting Information Tables S2). Except for two SCLCs, the H1184 cell line with *MYCL1* amplification and the SM09-011T1 tumor with *MYC* amplification, amplification of genes defined by 250K SNP arrays was consistently detected by real-time genomic-PCR (Supporting Information Table S3). On the other hand, in seven SCLCs without amplification by 250K SNP array analyses, 1–6 genes were judged as being amplified by real-time genomic-PCR analyses. Inconsistencies of the results were due to the following reasons. Firstly, only a small region including *MYC* covered by two SNP markers was amplified in the Lu135 cell line; therefore, this region was not defined as being amplified by SNP array analysis, but defined as being amplified by real-time genomic-PCR analysis. Secondly, in R-511T and SM09-006T, copy number loss of the reference locus, *RPPH1*, on chromosome 14 enhanced the degree of amplification in several genes by real-time genomic-PCR analysis. Thirdly, in H2195 and R-506M1, it was difficult to define the copy numbers possibly due to the heterogeneity of aneuploid cells and the presence of contaminated non-cancerous cells, respectively. Therefore, in these SCLCs, genes with copy number ratios ≥3 by real-time genomic-PCR was defined as five copies by SNP array analysis.

Even with some inconsistencies between the results of SNP array analyses and those of real-time genomic-PCR analyses, the association between them was highly significant (*P* = 5.58E-07 by Fisher’s exact test). Therefore, we then investigated the prevalence and specificity of gene amplification based on the data obtained by real-time genomic-PCR analysis. Amplification of these genes was detected in 12 of the 62 fresh tumors (19.4%) and 13 of the 25 cell lines (52.0%) (Table 2). Three *MYC* family genes were amplified in a mutually exclusive manner in 16 SCLCs (18.4%). Genes on chromosome 9p were amplified in 10 SCLCs (11.5%). Notably, *NF1B/FLJ41200* at 9p23 and *JAK2/CD274/PLGRKT/KIAA1432* at 9p24.21 were not co-amplified in 8 of the 10 SCLCs, indicating that amplification of these two regions occurred independently in most SCLCs. Therefore, the presence of target genes for amplification in each region was highly suggested. For this reason, specificities of 9p23 amplification and 9p24.21 amplification were independently analyzed among the 87 SCLCs. Importantly, four genes in the 9p24.1 region were amplified in a mutually exclusive manner with *MYC* family genes, whereas the *MYC* gene was co-amplified in one of three SCLCs with *NF1B* amplification, consistent with the results of 250K SNP array analyses (Supporting Information Fig. S2).

**TABLE 2:** Occurrence of Gene Amplification in a Mutually Exclusive Manner in Small Cell Lung Cancers

	1p34.2	2p24.3	8q24.21	9p23		9p24.1			
Sample name	*MYCL1*	*MYCN*	*MYC*	*NFIB*	*FLJ41200*	*JAK2*	*PLGRKT*	*CD274*	*KIAA1432*
H1963	+	–	–	–	–	–	–	–	–
HCC33	+	–	–	–	–	–	–	–	–
H510	+	–	–	–	–	–	–	–	–
H2141	+	–	–	–	–	–	–	–	–
SM09-012T	+	–	–	–	–	–	–	–	–
S391T	+	–	–	–	–	–	–	–	–
H69	–	+	–	–	–	–	–	–	–
H526	–	+	–	–	–	–	–	–	–
1591T	–	+	–	–	–	–	–	–	–
R-511T	–	+	–	–	–	–	–	–	–
H82	–	–	+	–	–	–	–	–	–
N417	–	–	+	–	–	–	–	–	–
Lu135	–	–	+	–	–	–	–	–	–
H2171	–	–	+	–	–	–	–	–	–
SM09-019T	–	–	+	–	–	–	–	–	–
H446	–	–	+	+	+	–	–	–	–
H2195	–	–	–	+	+	–	–	–	–
SM09-008T	–	–	–	–	+	–	–	–	–
SM09-006T	–	–	–	+	+	+	+	+	+
H1607	–	–	–	–	+	+	+	+	+
R-506M1	–	–	–	–	–	+	+	+	+
SM09-010T	–	–	–	–	–	–	+	+	+
R-513T	–	–	–	–	–	–	+	+	+
SM09-004T	–	–	–	–	–	–	+	+	–
1491M	–	–	–	–	–	–	–	–	+
Amplification rate in 87 SCLCs (%)	6.9	4.6	6.9	3.4	5.7	3.4	6.9	6.9	6.9

### Identification of Fusion Transcripts by Whole-transcriptome Sequencing

Forty-two SCLCs, consisted of 19 fresh tumors and 23 cell lines, were subjected to whole-transcriptome sequencing to identify fusion genes expressed in SCLCs (Supporting Information Tables S1A, B and S4). Total read counts ranged from 74,378,482 to 93,612,490, and their average was 86,529,758. A total of 60 fusion transcripts, transcribed from a portion of 95 genes, were identified as being expressed by the criteria of ≥10 paired-end (PE) reads for each transcript (Table 3). Twenty-two of them (36.7%) were in-frame; thus, were predicted to produce fusion proteins. There was no set of 5′-3′ fusion transcript recurrently detected in ≥2 SCLCs. However, two of the 5′ partner genes, *PVT1* and *RLF*, were detected recurrently in ≥2 SCLCs (14 pairs in 7 SCLCs). *PVT1* was detected as the 5′ partner gene of seven fusion pairs with different 3′ partner genes in five SCLCs. Previously, *PVT1* was shown to be fused with *CHD7* in H2171 and Lu135 (Campbell et al., 2008; Pleasance et al., 2010). In this study, *PVT1-CHD7* was detected in H2171 with ≥10 PE reads but was not in Lu135. *RLF* was detected as the 5′ partner gene of 7 fusion pairs with different 3′ partner genes in 2 SCLCs. *RLF* was reported as being a fusion gene with *MYCL1* expressed in SCLC cells (Makela et al., 1991a, 1991b, 1995), and the *RLF-MYCL1* fusion was also detected in H1963 with ≥10 PE reads in this study. Three of the five fusion pairs having *RLF* as the 5′ partner gene, *RLF-MYCL1*, *RLF-SMAP2* and *RLF-FAM132A*, were predicted to produce fusion proteins. The remaining 46 fusion transcripts were detected in a single SCLC, respectively, and 19 of them were predicted to produce fusion proteins. Therefore, none of the 22 fusion transcripts predicted to produce fusion proteins was expressed recurrently in multiple SCLC cases.

**TABLE 3:** Fusion Transcripts Expressed in Small Cell Lung Cancer Cells Identified by Whole-transcriptome Sequencing

				5’ partner gene				3’ partner gene					Frame
No	Sample	#PE reads	#junction reads	Gene	mRNA No	Chr	Amp.	Gene	mRNA No	Chr	Amp.	Distance on genome (bp)	In frame
I. 5’-transcripts detected in ≥ 2 cases													
1	H82	408	239	*PVT1*	NR_003367.1	8	+	*MYH7*	NM_000257.2	14	+		
2	H2171	345	177	*PVT1*	NR_003367.1	8	+	*CHD7*	NM_017780.3	8	+	67027313	
3	H2171	219	20	*PVT1*	NR_003367.1	8	+	*SLC7A7*	NM_001126105.2	14		105507749	
4	H2171	114	17	*PVT1*	NR_003367.1	8	+	*CCNB1IP1*	NM_021178.3	14		NA	
5	H2107	37	13	*PVT1*	NR_003367.1	8		*NOL4*	NM_001198546.1	18		NA	
6	N417	34	82	*PVT1*	NR_003367.1	8	+	*CLVS1*	NM_173519.2	8	+	66392576	
7	H446	12	26	*PVT1*	NR_003367.1	8	+	*LY6H*	NM_001130478.1	8		15125833	
8	HCC33	525	128	*RLF*	NM_012421.3	1	+	*UBE2J2*	NM_058167.2	1		39417806	
9	H1963	391	562	*RLF*	NM_012421.3	1	+	*MYCL1*	NM_001033081.2	1	+	259353	+
10	H1963	194	68	*RLF*	NM_012421.3	1	+	*COL9A2*	NM_001852.3	1	+	59570	
11	H1963	118	68	*RLF*	NM_012421.3	1	+	*BCL2L1*	NM_001191.2	20	+	NA	
12	H1963	112	124	*RLF*	NM_012421.3	1	+	*HM13*	NM_030789.2	20	+	NA	
13	H1963	43	163	*RLF*	NM_012421.3	1	+	*SMAP2*	NM_001198978.1	1	+	133135	+
14	HCC33	33	132	*RLF*	NM_012421.3	1	+	*FAM132A*	NM_001014980.2	1		39444938	+
II. Fusion transcripts detected in a single case													
15	H1963	2168	1627	*TPX2*	NM_012112.4	20	+	*HM13*	NM_030789.2	20	+	169703	+
16	SM09-012T	290	335	*CAP1*	NM_001105530.1	1	+	*MACF1*	NM_012090.4	1	+	991332	
17	H1963	226	52	*BCL2L1*	NM_138578.1	20	+	*HM13*	NM_030789.2	20	+	94884	
18	HCC33	193	83	*TRIT1*	NM_017646.4	1	+	*EP400*	NM_015409.4	12	+	NA	
19	H1963	125	45	*BCL2L1*	NM_138578.1	20	+	*DEM1*	NM_022774.1	1	+	NA	+
20	H2171	122	56	*ENO2*	NM_001975.2	12		*ACRBP*	NM_032489.2	12			+
21	H1963	99	99	*BCL2L1*	NM_138578.1	20	+	*RIMS3*	NM_014747.2	1	+	NA	
22	SM09-004T	82	101	*WAC*	NM_016628.4	10		*GPR158*	NM_020752.2	10		2931268	
23	Lu139	79	45	*CSMD3*	NM_052900.2	8		*MYC*	NM_002467.4	8		14299072	
24	H1184	54	88	*RERE*	NM_001042681.1	1		*SLC2A5*	NM_001135585.1	1		223728	+
25	H69	50	44	*FOXK2*	NM_004514.3	17		*HEXDC*	NM_173620.2	17		77073	
26	H1963	50	17	*BCL2L1*	NM_138578.1	20	+	*ZNF684*	NM_152373.3	1	+	NA	+
27	N417	42	30	*NCOR2*	NM_001077261.3	12		*SCARB1*	NM_005505.4	12		210164	
28	HCC33	41	71	*UBE4B*	NM_001105562.2	1		*TBCB*	NM_001281.2	19		NA	+
29	SM09-010T	41	43	*KIAA1432*	NM_001135920.2	9	+	*JAK2*	NM_004972.3	9		501144	+
30	H1963	41	31	*ZMPSTE24*	NM_005857.4	1	+	*MFSD2A*	NM_001136493.1	1	+	288105	
31	H510	38	37	*SMEK1*	NM_032560.4	14		*HEATR3*	NM_182922.2	16		NA	+
32	SM09-016T	36	48	*NAV2*	NM_001111018.1	11		*LOC494141*	NR_026563.1	11		1137161	
33	Lu135	32	23	*LRRC45*	NM_144999.2	17		*GCGR*	NM_000160.3	17		209390	
34	H510	30	67	*TWSG1*	NM_020648.5	18		*PIK3C3*	NM_002647.2	18		30132781	
35	H69	30	34	*PAWR*	NM_002583.2	12		*GNS*	NM_002076.3	12		14832520	
36	H1184	25	20	*SF3A3*	NM_006802.2	1	+	*GNL2*	NM_013285.2	1	+	361067	+
37	H209	24	19	*CREBBP*	NM_001079846.1	16		*SLX4*	NM_032444.2	16		113472	
38	SM09-016T	24	14	*RASA2*	NM_006506.2	3		*NICN1*	NM_032316.3	3		91739168	+
39	H1963	22	48	*SMAP2*	NM_022733.2	1	+	*MYCL1*	NM_001033081.2	1	+	472040	
40	SM09-016T	22	13	*SFMBT1*	NM_001005158.2	3		*AP2A2*	NM_001242837.1	11		NA	
41	H510	21	12	*PTK2*	NM_001199649.1	8		*PKHD1L1*	NM_177531.4	8		31125439	
42	H526	19	31	*SLC25A36*	NM_001104647.1	3		*PLSCR1*	NM_021105.2	3		5534191	
43	H526	19	20	*XPR1*	NM_001135669.1	1		*TRMT1L*	NM_001202423.1	1		4227805	+
44	H2195	18	22	*HMBOX1*	NM_001135726.1	8		*ZFAND3*	NM_021943.2	6		NA	+
45	SM09-014T	18	10	*CRLS1*	NM_001127458.1	20		*KCNK17*	NM_001135111.1	6		NA	
46	H510	17	14	*PHF15*	NM_015288.4	5		*UBE2B*	NM_003337.2	5		133999	
47	H1963	17	10	*BCL2L1*	NM_138578.1	20	+	*ZNF643*	NM_023070.2	1	+	NA	+
48	SM09-016T	15	40	*ATP5L*	NM_006476.4	11		*TEAD1*	NM_021961.5	11		105305820	
49	SM09-014T	14	23	*NUDCD1*	NM_001128211.1	8		*SYBU*	NM_001099743.1	8		244247	+
50	Lu134	14	17	*SPG11*	NM_001160227.1	15		*SORD*	NM_003104.5	15		359425	+
51	SM09-016T	14	17	*NGLY1*	NM_001145293.1	3		*CCKBR*	NM_176875.3	11		NA	+
52	SM09-016T	13	21	*DLEC1*	NM_007335.2	3		*ODZ4*	NM_001098816.2	11		NA	
53	H1963	13	19	*PPT1*	NM_000310.3	1	+	*BCL2L1*	NM_001191.2	20	+	NA	
54	H128	13	12	*NAIP*	NM_004536.2	5		*OCLN*	NM_001205254.1	5		1414179	
55	H1963	12	10	*BCL2L1*	NM_138578.1	20	+	*BMP8B*	NM_001720.3	1	+	NA	
56	H526	12	10	*CIT*	NM_001206999.1	12		*RFC5*	NM_001130112.2	12		1653559	+
57	H1963	11	22	*BCL2L1*	NM_138578.1	20	+	*RLF*	NM_012421.3	1	+	NA	
58	SM09-018T	10	22	*NFIX*	NM_002501.2	19		*GATAD2A*	NM_017660.3	19		6287032	+
59	SM09-016T	11	19	*STAG1*	NM_005862.2	3		*STXBP5L*	NM_014980.2	3		14912391	
60	H2171	10	12	*PICALM*	NM_001008660.2	11		*CCDC81*	NM_001156474.1	11		304853	+

### Amplification of Genes with Fusions

We next investigated the copy numbers of 95 genes in the 60 fusion pairs identified by whole-transcriptome sequencing (Table 3). Twenty-eight of the 5′ partner genes detected in 9 SCLCs and 22 of the 3′ partner genes detected in seven SCLCs were mapped in the amplified regions.

In four of the five SCLCs expressing fusion transcripts with *PVT1* as the 5′ partner gene, the 5′ portions of the *PVT1* gene at 8q24.21 were amplified, indicating that chromosomal breaks had occurred in the *PVT1* locus (Supporting Information Fig. S1). Three of seven genes fused with *PVT1* were also amplified. Therefore, it was indicated that chromosomal breaks often occur in the *PVT1* locus during the process of *MYC* amplification. For this reason, we further searched for *PVT1* fusion transcripts expressed in the H2171, Lu135, and N417 cell lines, which showed co-amplification of three regions on chromosome 8q (Table 1). In addition to *PVT1-CHD7* (PE reads = 345), *PVT1-SLC7A7* (PE read = 219), and *PVT1-CCNB1IP1* (PE reads = 114) were detected in H2171. As described above, no *PVT1* fusion was detected in Lu135. *PVT1-CLVS1* (PE read = 34) and *PVT1-ASPH* (PE reads = 27, junction reads = 9) were detected in N417.

*RLF,* detected as the 5′ partner gene of seven fusion pairs in two cell lines, H1963 and HCC33, was amplified in both cell lines (Table 3). Three 3′ partner genes, *MYCL1*, *COL9A2,* and *SMAP2*, fused with *RLF* in H1963 were also mapped to 1p34.2 and amplified. Two other 3′ partner genes, *BCL2L1* and *HM13*, fused with *RLF* in H1963 and mapped to 20q11.21, were also amplified. The remaining two 3′ partner genes, *UBE2J2* and *FAM132A* at 1p36.33, were also amplified in HCC33 with consecutive 2 SNPs. Therefore, production of fusion transcripts with *RLF* was always accompanied by amplification of both the 5′ and 3′ genes, indicating that those genes had fused in the process of *MYCL1* amplification.

These results strongly indicate that amplification of several regions on chromosomes 1 and 8 simultaneously but not sequentially occurs in SCLC cells, and further support that complicated intrachromosomal rearrangements occur in the process of *MYCL1* or *MYC* amplification, resulting in the co-amplification and fusion of several genes on chromosomes 1 and 8. Therefore, the *PVT1* and *RLF* loci would be hotspots of chromosomal breaks in the process of gene amplification in SCLC cells.

### SCLCs with Expression of Multiple Fusion Transcripts

Sixty fusion transcripts were detected in 23 of the 42 SCLCs (Table 3, Supporting Information Table S4), indicating the presence of SCLCs expressing multiple fusion transcripts. Indeed, sixteen fusion pairs consisted of 15 genes were identified in H1963 (Supporting Information Fig. S4A). Twelve of the 15 genes, including *MYCL1*, were mapped to the 1p34.2 amplicon, and the remaining 3 genes were mapped to the 20q11.21 amplicon. Seven fusions were intrachromosomal among genes at 1p34.2 or 20q11.21, while the other nine fusions were interchromosomal between genes at 1p34.2 and genes at 20q11.21. Therefore, in H1963, complicated chromosomal rearrangements were likely to have occurred in the process of *MYCL1* amplification.

Seven fusion pairs consisted of 14 genes were identified in SM09-016T (Supporting Information Fig. S4B). Seven and 7 of the 14 genes were mapped to chromosomes 3 and 11, respectively. The 5′ and 3′ partner genes for four of the seven fusions were mapped to the same chromosomes, while those for the remaining three fusions were mapped to different chromosomes. Therefore, these genes were fused by either intrachromosomal or interchromosomal rearrangements. Interestingly, no fused genes were amplified in SM09-016T, indicating that complicated chromosomal rearrangements had occurred without gene amplification. Two to five fusion transcripts were detected in eight other SCLCs. Among 24 fusions detected in these SCLCs, 18 of them were intrachromosomal and the remaining six were interchromosomal. Eight of 5′ partner genes and four of 3′ partner genes were mapped to the amplified regions. Therefore, intrachromosomal rearrangements seemed to occur preferentially in SCLC cells irrespective of the process of gene amplification.

### *KIAA1432-JAK2* Fusion Detected in a SCLC with 9p24.1 Amplification

**Figure 3:**
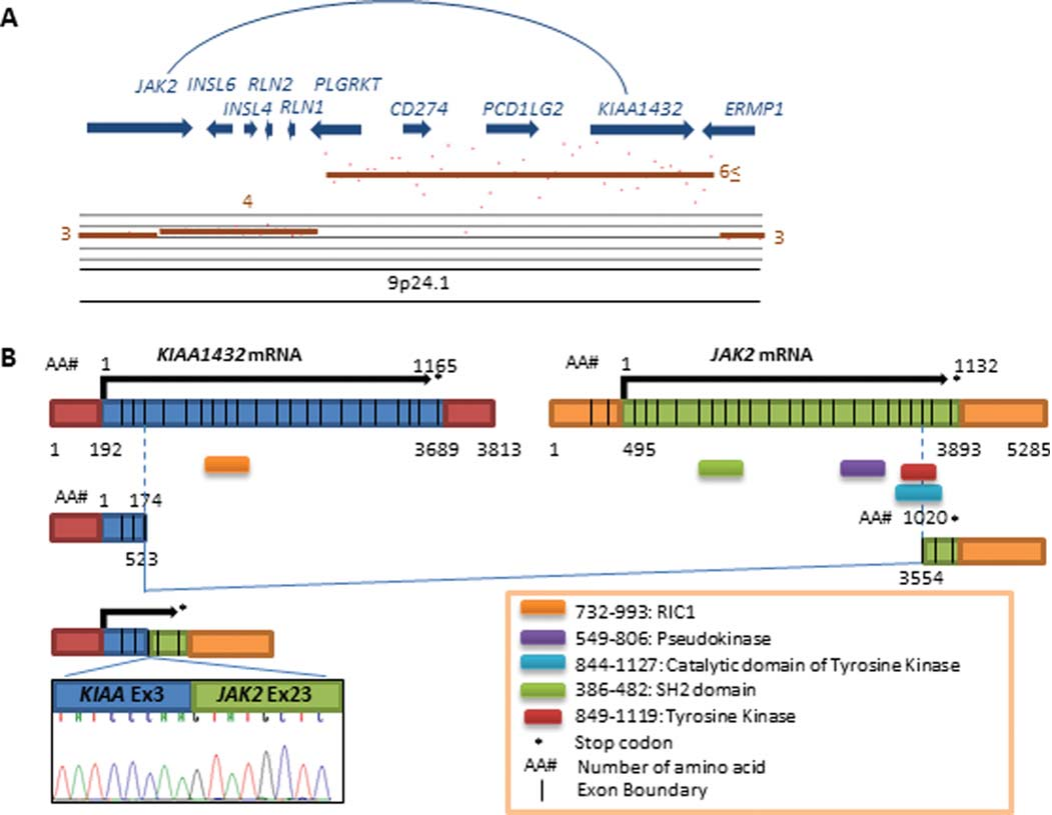
A *KIAA1432-JAK2* fusion detected in the SM09-010T tumor with 9p24.21 amplification. (A) Copy number plots at 9p24.1 by 250K SNP array. (B) Schematic presentation of wild-type *KIAA1432* and *JAK2* proteins, and the *KIAA1432-JAK2* fusion protein. Electrophoregram for Sanger sequencing of cDNA for the *KIAA1432–JAK2* fusion transcript is shown below.

Interestingly, a *KIAA1432–JAK2* fusion transcript was detected in SM09-010T with amplification of the *KIAA1432* gene at 9p24.1 (Table 3; Fig. 3A). Furthermore, three (4.6%) of 65 SCLCs analyzed by RT-PCR were shown to express *KIAA1432–JAK2* fusion transcripts. However, only one of them expressed in SM09-010T was predicted to produce a fusion protein, although the tyrosine kinase domain was disrupted by fusion (Fig. 3B).

## DISCUSSION

The purpose of this study was to identify genes activated by amplification and/or fusion in SCLC. By a copy number analysis, 34 genes were identified as being frequently amplified in SCLCs. In concordance with previous studies, three *MYC* family genes were frequently amplified in SCLCs (Wistuba et al., 2001; Kim et al., 2006; Voortman et al., 2010; Larsen and Minna, 2011; Sos et al., 2012). Recently, several MYC inhibitors, including Omomyc, BET bromodomain inhibitor and Aurora kinase inhibitor, have been reported (Soucek et al., 2008; Delmore et al., 2011; Sos et al., 2012); therefore, the MYC family gene products could be druggable targets in SCLC cells with their activation.

Co-amplification of *PVT1* and *MIR1204* with *MYC* has been reported in several types of cancers, and their oncogenic roles have been also suggested (Guan et al., 2007; Huppi et al., 2008; Haverty et al., 2009; Schiffman et al., 2010; Sircoulomb et al., 2010). However, in this study, only the 5′ portion of the *PVT1* gene including exon 1 was commonly amplified, and amplified *PVT1* genes were often fused with other genes. Since the 3′ partner genes in five *PVT1* fusions were different from each other, biological significance of *PVT1* amplification and/or fusion is unclear at present. The *PVT1* locus could be a hotspot of chromosomal breaks in the process of *MYC* amplification in SCLCs. Since *MIR1204* was always co-amplified with *MYC*, involvement of *MIR1204* in the development of SCLC cannot be excluded. Recently, *PVT1-MYC* fusions were detected in >60% of medulloblastomas with *MYC* amplification, and these fusions also involved *PVT1* exon1 and *MIR1204* (Northcott et al., 2012). These results further support that the *PVT1* locus is a hotspot of chromosomal breaks in the process of *MYC* amplification, although no *PVT1-MYC* fusions were detected in SCLCs.

In addition to *MYCL1* and *MYC*, several regions on either the same or different chromosomes were co-amplified in SCLCs. Furthermore, several genes, especially *RLF* and *PVT1* in the *MYCL1* and *MYC* amplicons, respectively, were fused with genes in different amplified regions. These results strongly indicate that amplification of several regions on chromosomes 1 and 8 occurred simultaneously but not independently/sequentially in these SCLCs. Therefore, several genes co-amplified with *MYCL1* and *MYC* were likely to be rearranged and amplified together with *MYCL1* and *MYC* by a massive genomic rearrangement acquired in a single catastrophic event. Recently, a new mechanism for genetic instability in cancer cells, chromothripsis, was proposed by Stephens et al. (2011). In chromothripsis, tens to hundreds of chromosomal rearrangements involving localized genomic regions can be acquired in a one-off cellular catastrophe. Indeed, *CHD7* at 8q12 was shown to be rearranged in three SCLC cell lines (Campbell et al., 2008; Pleasance et al., 2010). In this study, amplified genes in SCLC cells often showed fusions with genes in the same amplicons, different amplicons on the same chromosome, or different amplicons on different chromosomes. These results strongly indicate that amplification of *MYCL1* and *MYC* often occurs through chromothripsis in SCLCs, although the presence of tens to hundreds of chromosomal rearrangements in particular genomic regions should be confirmed by whole genome sequencing. Therefore, target genes of amplification on these chromosomes would be *MYCL1* and *MYC*, respectively, even though multiple regions on chromosomes 1 and 8 were commonly amplified in SCLCs.

Two regions on chromosome 9p were also commonly amplified in SCLCs. Notably, amplification at 9p24.1 tended to occur in SCLCs without amplification of *MYC* family genes. In contrast, the 9p23 region including *NF1B* was co-amplified with *MYC* in H446. Previously, *Nf1b* in a mouse SCLC model was shown to be frequently co-amplified with *Mycl1* (Dooley et al., 2011), consistent with the present results. However, 9p24.1 and 9p23 were independently amplified in most SCLCs. Therefore, these regions were unlikely to be amplified by chromothripsis, and the 9p23 and 9p24.1 regions may contain independent target genes, respectively. Expression analyses revealed that *NF1B* at 9p23 and *KIAA1432* at 9p24.1 were overexpressed by gene amplification in SCLCs, thus, were strong candidates of genes activated by amplification in SCLCs. Recently, *KIAA1432* was reported to be also amplified and overexpressed in breast cancer, thus, is a target gene of amplification not only in SCLC but also in breast cancer (Wu et al., 2012). *KIAA1432* encodes a partner protein, CIP150, of connexin 43 (Cx43) (Akiyama et al., 2005). Cx43, a structural protein in the gap junction, has been reported as being a tumor suppressor inactivated in several cancers (Li et al., 2008; Naus and Laird, 2010; Plante et al., 2011). Therefore, it is possible that CIP150 encoded by *KIAA1432* is involved in the regulation of Cx43 activities and its overexpression may play a role in SCLC development. Further functional studies are needed to clarify the biological significance of *KIAA1432* amplification in SCLC development. Interestingly, a *KIAA1432-JAK2* fusion was identified in a case with *KIAA1432* amplification. Various fusions with *JAK2* have been reported in hematological malignancies (Van Roosbroeck et al., 2011). These fusions contained the whole tyrosine kinase domain and lead to constitutive phosphorylation of the kinase (Lacronique et al., 1997; Griesinger et al., 2005; Poitras et al., 2008; Nebral et al., 2009; Van Roosbroeck et al., 2011). However, the kinase domain was disrupted by the *KIAA1432-JAK2* fusion identified in this study. Therefore, it is unlikely that *JAK2* is activated by fusion with *KIAA1432* in SCLCs. There might be hotspots of chromosomal breakpoints in the *JAK2* and *KIAA1432* loci in the process of 9p24.1 amplification in SCLCs.

During the preparation of this manuscript, the results of comprehensive and integrative genome analyses on SCLCs were reported by two groups (Peifer et al., 2012; Rudin et al., 2012). Frequent amplification of the *SOX2* (copy number ≥4) and *FGFR1* (copy number ≥3.5) genes at 3q26.3-q27 and 8p12, respectively, were shown in their articles. When we used the same criteria (copy number ≥4), the *SOX2* and *FGFR1* genes were amplified in 21 (36.0%) and 7 (12.1%), respectively, of 58 SCLCs subjected to 250K SNP array analysis. However, in this study, by using the criteria of copy number ≥6 for detection of focally amplified genes, neither *SOX2* nor *FGFR1* were picked up as the amplified genes in SCLCs, because extents of amplification for the *SOX2* and *FGFR1* genes were not so high as those for *MYC* family genes and 9p genes. Therefore, in our criteria, genes with activation by low degree of amplification (3–5 copies) were overlooked. However, genes with high degree of amplification (copy number ≥6) were successfully and efficiently picked up from the SCLC genomes. A recurrent *RLF-MYCL1* fusion was reported in one article (Rudin et al., 2012). The *RLF-MYCL1* fusion was also identified in H1963 in this study, but both *RLF* and *MYCL1* were fused with several other genes in this cell line, indicating the occurrence of chromothripsis in the production of those fusions in SCLCs.

We should also point out here that statuses of *MYC* family gene amplification in some cell lines defined in this study were not the same as those reported previously. To depict such inconsistencies more critically and clearly, we prepared Supporting Information Table S5, in which the statuses for *MYC* family amplification defined in this study were summarized together with those in three other studies (Kim et al., 2006; Voortman et al., 2010; Sos et al., 2012) for each of all the 25 cell lines analyzed in this study. In 18 of the 25 cell lines, statuses of *MYC* family gene amplification were also defined in 1–3 of the other studies. In 14 of the 18 cell lines, statuses of *MYC* family gene amplification were consistent among studies. However, in the H69 cell line, *MYCN* amplification was detected in three of the four studies, and in the remaining three cell lines, H128, H187, and H2107, either *MYC* or *MYCL1* amplification was detected only in one of three or four studies. These inconsistencies would be due to the differences in the criteria of gene amplification among the four studies and also could be due to the differences in the methods as well as the platforms used for assessing copy numbers of each gene among them.

In this study, we did not refer to somatic mutations that could be detected by whole-transcriptome sequencing, because genes with somatic mutations that are highly expressed in the cells can be only detected by whole-transcriptome sequencing. In our preliminary results, various types of mutations detected by genome sequencing were not detected by whole transcriptome sequencing possibly due to the low levels or absence of expression. In addition, due to the differences in the level of mRNA expression among genes analyzed, total read counts of transcripts for sequencing varied among genes in each sample. Therefore, it was difficult to obtain conclusive results for the presence of mutations by whole-transcriptome sequencing only. To obtain more convincing results, we have to confirm the presence of mutations by using several types of genome sequencing, such as direct sequencing and whole exome/genome sequencing. Accordingly, in this study, we did not present the data for possible somatic mutations detected by whole-transcriptome sequencing. In contrast, the presence of amplified or fused genes could be easily confirmed by PCR analysis. Therefore, in this study, we attempted to compile the list of genes that were activated by amplification and/or fusion in SCLC cells. Further studies are now in progress.
