# Supplementary material for: Genome-wide identification of genes with amplification and/or fusion in small cell lung cancer
Source: Genes Chromosomes Cancer. 2013 May 28;52(9):802–16. doi: 10.1002/gcc.22076 (PMC3806277; doi:10.1002/gcc.22076)
Supplement: Supplementary file 1 [file gcc0052-0802-sd1.docx]

**A Fresh tumors**

**Supplementary Table S1. List of SCLC samples and primers for real-time genomic-/RT-PCR used in this study**


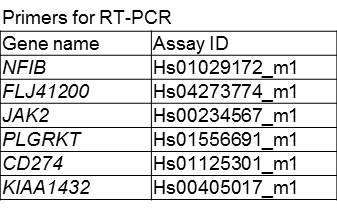

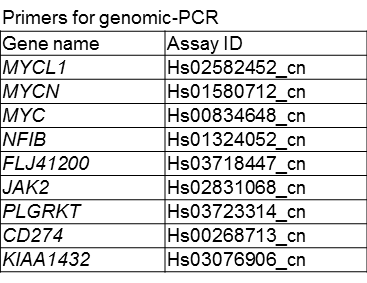


All primers were purchased from Applied Biosystems (ABI).

**C Primers for real-time genomic-/RT-PCR**

**B Cell lines**

**Supplementary Table S1. -continue**


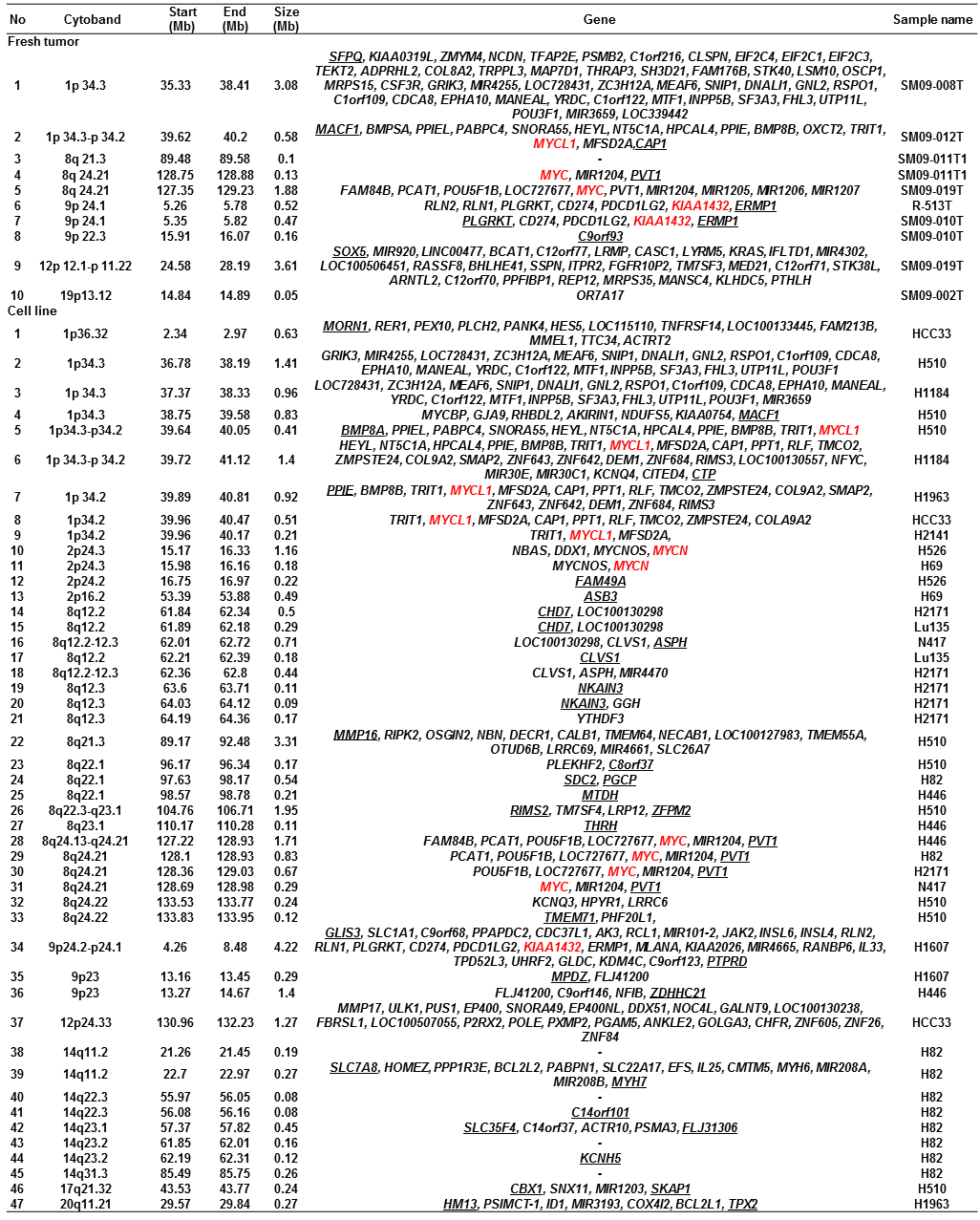


**Supplementary Table S2. Chromosomal regions and genes highly amplified in 33 fresh tumors and 25 cell lines**

Genes with copy number alteration inside them are underlined. Candidate target genes are marked in red.


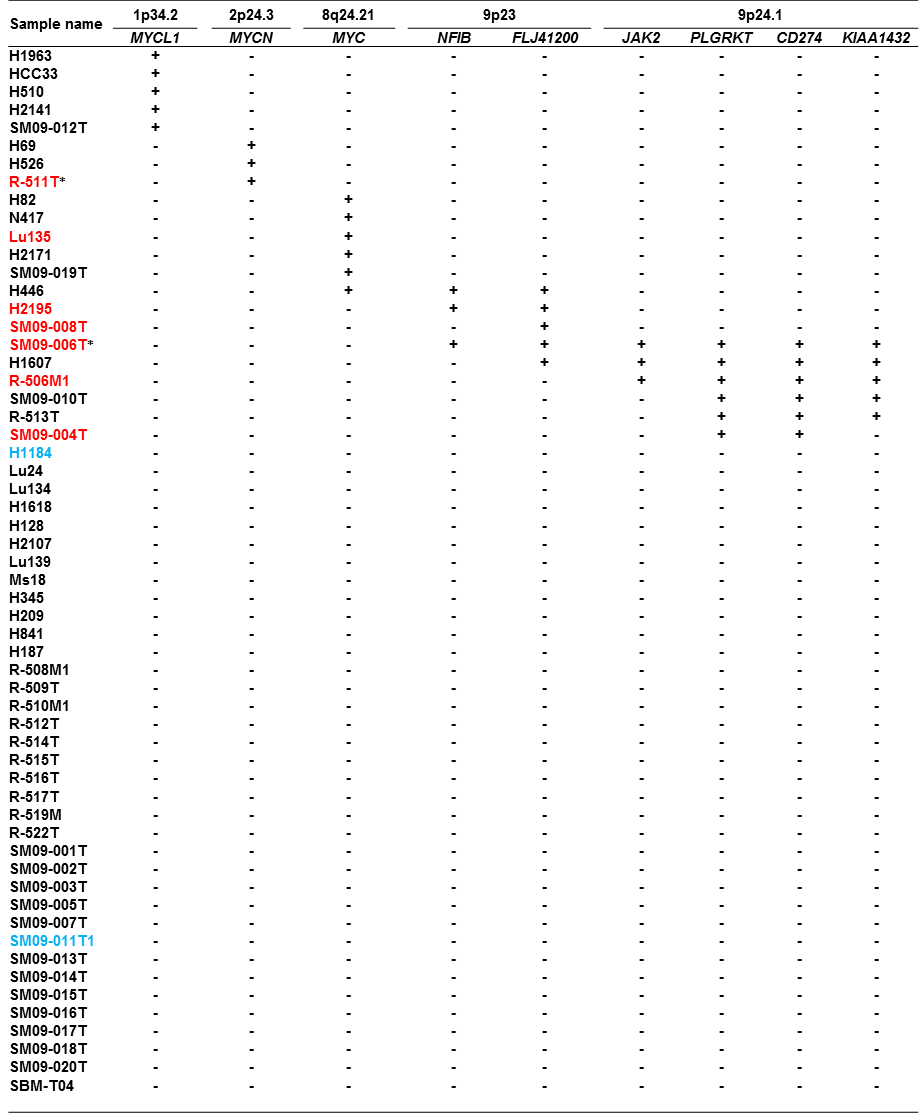


+: amplified (Gene/RPPH1 ratio ≥ 3.0), -: not amplified (Gene/RPPH1 ratio < 3.0) *****SCLCs with an estimated the copy number of one in RPPH1. SCLCs marked in red showed amplification by real-time genomic-PCR analysis but not by 250K SNP array analysis. SCLCs marked in blue did not show amplification by real-time genomic-PCR analysis but showed by 250K SNP array analysis.

**Supplementary Table S3. Consistency for the results of real-time genomic-PCR with those of 250K SNP array analysis**

42 SCLCs, consisted of 23 cell lines and 19 fresh tumors, subjected to whole-transcriptome sequencing are listed in this table.

**Supplementary Table S4. Number of fusion transcripts detected in each SCLC case**

-： no MYC family gene amplification, blank: not done.

**Supplementary Table S5. Status of MYC family gene amplification in SCLC cell lines**


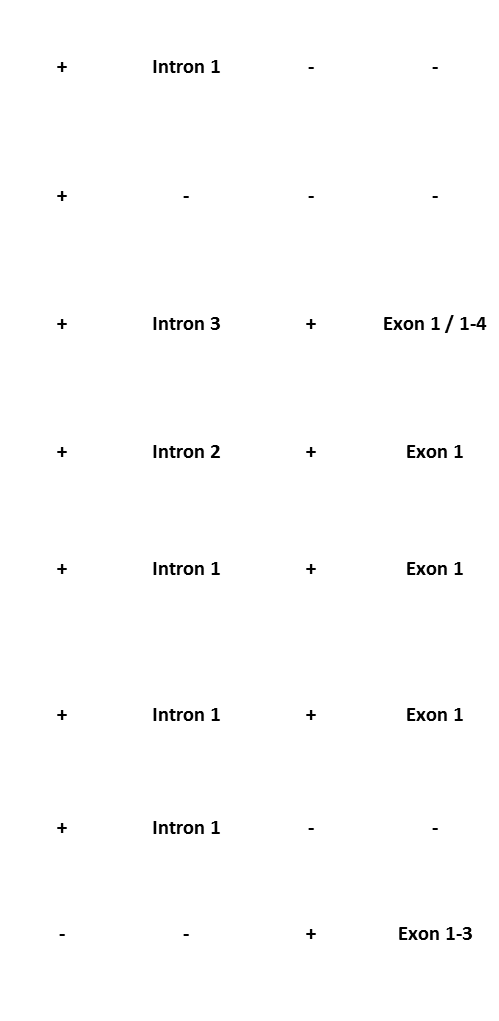

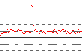

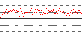

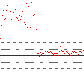

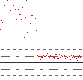

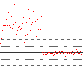

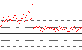

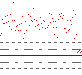

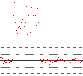


**The 5’ end of the *PVT1* gene**

**Supplementary Figure S1. SCLCs with amplification and/or fusion of the *PVT1* gene**

**Amplification**

Copy number plots by 250K SNP array analysis of the 8 SCLCs with amplification and/or fusion of the *PVT1* gene are shown. Total copy numbers are indicated by red bars. Locations of *MYC*, *PVT1* and *MIR1204* are indicated by blue arrows or bar.

***MIR1204***

***MYC***

***PVT1***

**4**

**4**

**4**

**3**

**3**

**2**

**2**

**2**

**4**

**5**

**4**

**6 ≤**

**6 ≤**

**6 ≤**

**6 ≤**

**6 ≤**

**6 ≤**

6 ≤

**Exon(s) in fusion transcripts**

**Fusion**

**Break point**

**H2107**

**Lu135**

**H82**

**N417**

**N446**

**H2171**

**SM09-019T**

**SM09-011T1**


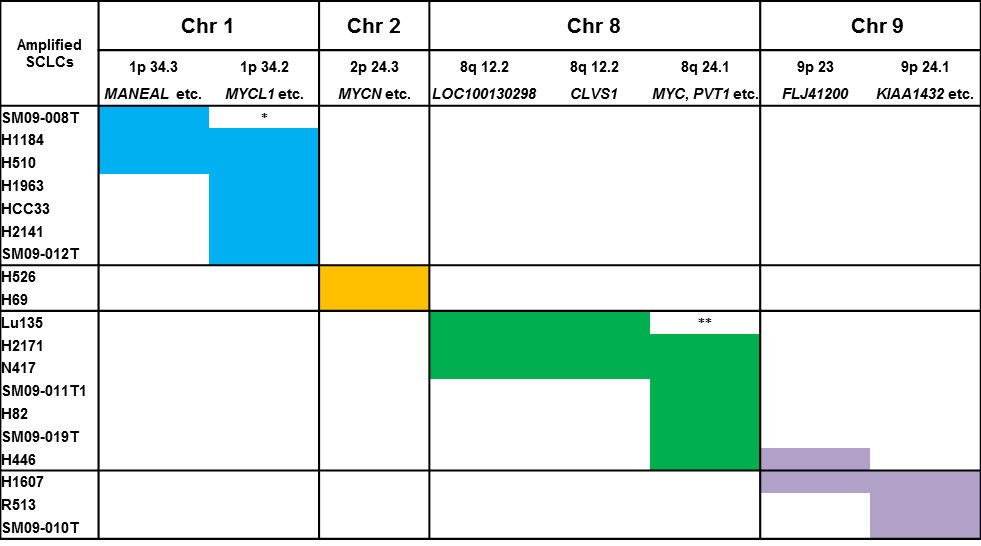


**Supplementary Figure S2. Occurrence of gene amplification in a mutually exclusive manner in small cell lung cancers**

Amplified regions in each SCLC are highlighted in colors. * The 1p34.2 region of SM09-008T was not judged as an amplified region (≥ 6 copies) but was a region of high copy number gain (5 copies). ** The 8q24.21 region of Lu135 was not judged as an amplified region, but a small region containing *MYC*, *PVT1* and *MIR1204* was amplified.


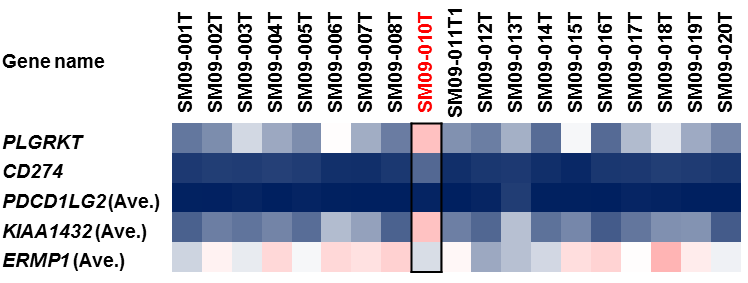

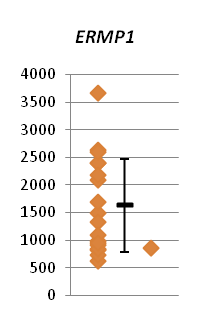

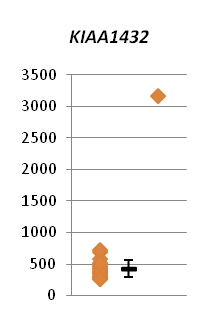

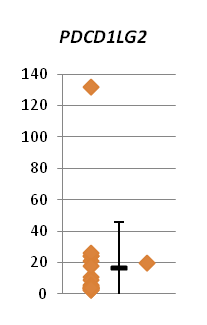

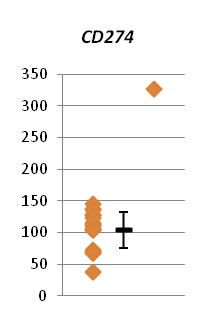

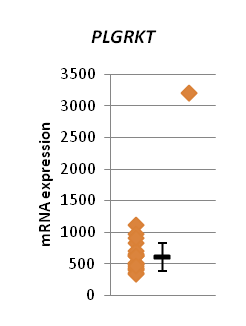


**Centromere**

**Telomere**

**Gene amplification**

**(+)**

**(-)**

**(+)**

**(-)**

**(+)**

**(-)**

**(+)**

**(-)**

**(+)**

**(-)**

A. Expression profiling data of genes mapped in the commonly amplified region at 9p24.1 are shown as a heatmap. SM09-010T described in red showed amplification of this region. B. Levels of mRNA expression by expression profiling in amplified (+) (N = 1) and not amplified (-) (N = 18) SCLCs are shown.

**B**

**A**

**Supplementary Figure S3. mRNA expression of genes in the commonly amplified region at 9p24.1 by expression profile analysis**


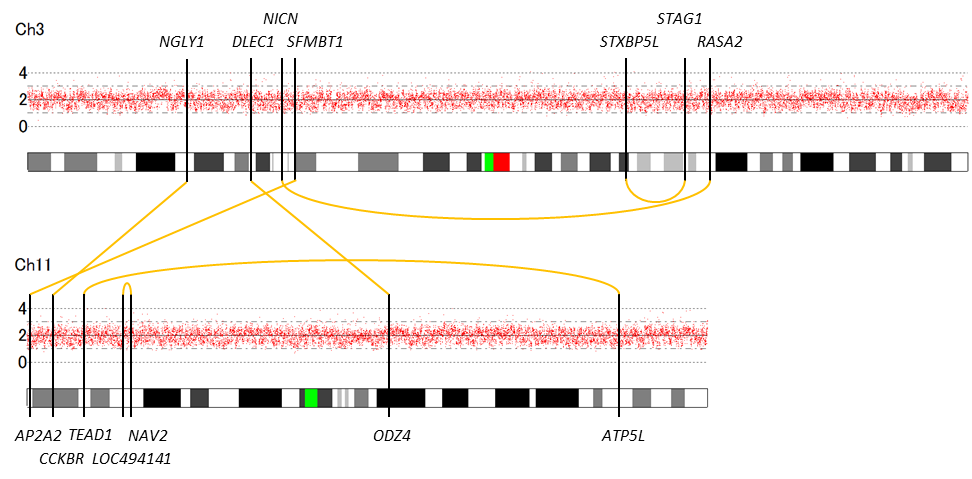

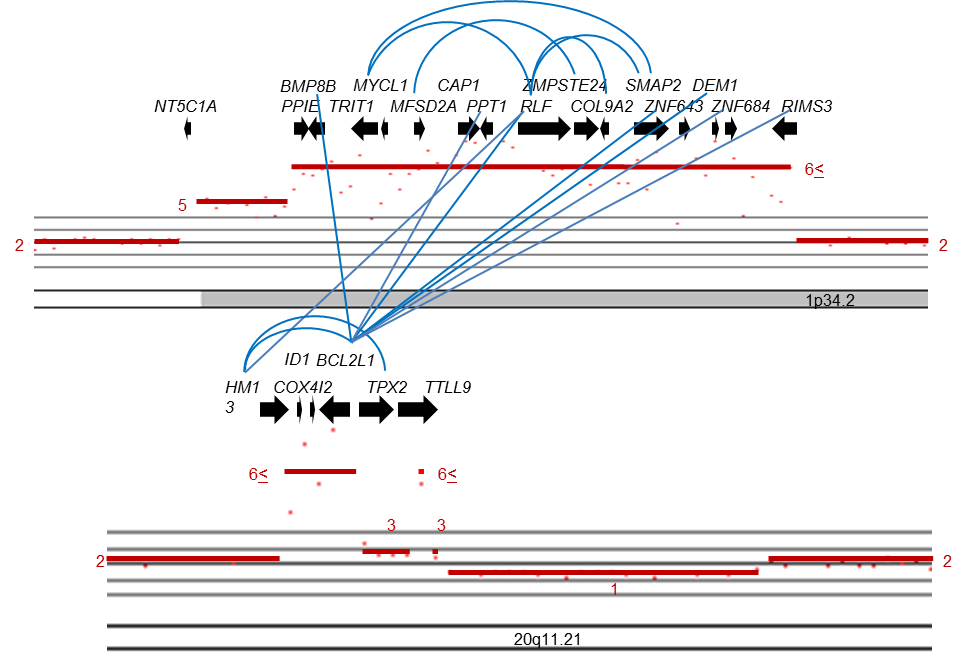


**B SM09-016T**

**A H1963**

Copy number plots by 250K SNP array and fusions detected with ≥10 PE reads in the H1963 cell line and the SM09-016T fresh tumor are shown. A. Total copy numbers are indicated by red bars, and genes mapped in these amplified regions are indicated with arrows. The 5’ and 3’ partner genes are connected by blue lines. Reciprocal fusions of *RLF-BCL2L1* and *BCL2L1-RLF* were detected with ≥10 PE reads as shown in Table 3. B. Locations of fusion genes are indicated by black lines, and the 5’ and 3’ partner genes are connected by yellow lines.

**Supplementary Figure S4. Fusions detected with ≥10 PE reads in the H1963 cell line and the SM09-016T fresh tumor**
